# Pili Torti: A Feature of Numerous Congenital and Acquired Conditions

**DOI:** 10.3390/jcm10173901

**Published:** 2021-08-30

**Authors:** Aleksandra Hoffmann, Anna Waśkiel-Burnat, Jakub Żółkiewicz, Leszek Blicharz, Adriana Rakowska, Mohamad Goldust, Małgorzata Olszewska, Lidia Rudnicka

**Affiliations:** 1 Department of Dermatology, Medical University of Warsaw, Koszykowa 82A, 02-008 Warsaw, Poland; aleksandraa.hoffmann@gmail.com (A.H.); kuba.zolkiewicz@gmail.com (J.Ż.); leszek.blicharz@wum.edu.pl (L.B.); adriana.rakowska@wum.edu.pl (A.R.); malgorzata.olszewska@wum.edu.pl (M.O.); lidia.rudnicka@wum.edu.pl (L.R.); 2 Department of Dermatology, University Medical Center of the Johannes Gutenberg University, 55122 Mainz, Germany; mgoldust@uni-mainz.de

**Keywords:** pili torti, trichoscopy, hair shaft abnormalities, hair shaft disorder, hair disease, twisted hair

## Abstract

Pili torti is a rare condition characterized by the presence of the hair shaft, which is flattened at irregular intervals and twisted 180° along its long axis. It is a form of hair shaft disorder with increased fragility. The condition is classified into inherited and acquired. Inherited forms may be either isolated or associated with numerous genetic diseases or syndromes (e.g., Menkes disease, Björnstad syndrome, Netherton syndrome, and Bazex-Dupré-Christol syndrome). Moreover, pili torti may be a feature of various ectodermal dysplasias (such as Rapp-Hodgkin syndrome and Ankyloblepharon-ectodermal defects-cleft lip/palate syndrome). Acquired pili torti was described in numerous forms of alopecia (e.g., lichen planopilaris, discoid lupus erythematosus, dissecting cellulitis, folliculitis decalvans, alopecia areata) as well as neoplastic and systemic diseases (such as cutaneous T-cell lymphoma, scalp metastasis of breast cancer, anorexia nervosa, malnutrition, cataracts, and chronic graft-vs.-host disease). The condition may also be induced by several drugs (epidermal growth factor receptor inhibitors, oral retinoids, sodium valproate, and carbamide perhydrate). The diagnosis of pili torti is based on trichoscopic or microscopic examination. As pili torti is a marker of numerous congenital and acquired disorders, in every case, the search for the signs of underlying conditions is recommended.

## 1. Introduction

**Figure 1:**
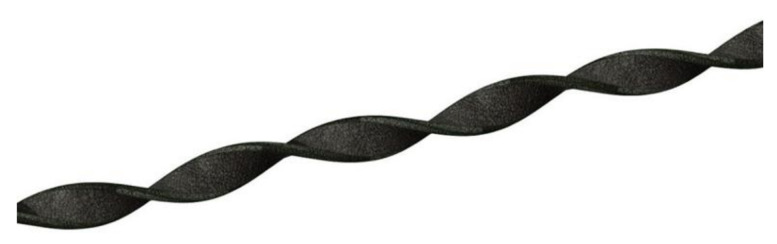
Pili torti, characterized by the presence of the hair shaft flattened and twisted 180° along its long axis. Reproduced with permission from J. Taczała, MSc.

Pili torti, also known as “twisted hair”, was first described by Galewsky, and, independently, by Ronchese in 1932 [1,2]. It is characterized by the presence of the hair shaft, flattened at irregular intervals and twisted 180° along its long axis, with each twist being 0.4 to 0.9 mm wide and occurring in groups of 3 to 10 (Figure 1) [3].

Pili torti is a form of hair shaft disorder with increased fragility [4]. It is classified into inherited or acquired. A wide range of changes associated with pili torti suggest specific pathophysiological mechanisms [5]. In inherited forms, the twisting of the hair is caused by an unequal development of the outer root sheath cells. Cell vacuolation and the irregular thickness of the outer root sheath at the suprabulbar level induce an uneven molding of the inner root sheath and hair shaft [6]. In acquired forms, a perifollicular inflammation followed by fibrosis generates rotational forces and deforms the hair follicle [7]. The cross-sectional area of pili torti hair is significantly smaller than a normal hair sample (2210 ± 1090 vs. 3370 ± 821 (µm^2^); *p* < 0.001) and the tensile strength of pili torti is 2.1 times lower than that of normal hair [8]. No abnormalities in the hair cortex keratin within pili torti axis are observed [5].

Clinically, patients with pili torti have fragile, brittle, dry, and coarse hair. Patchy alopecia may develop. The scalp hair, especially in the occipital and temporal areas, is most commonly affected [9]. However, the eyebrows, eyelashes, axillary, and pubic hair may also be involved [9]. Usually, not all hair is affected by pili torti, and only a part of hair length may be changed [10]. Isolated pili torti may be occasionally found in the normal scalp. However, it may be associated with numerous local and systemic conditions [11,12].

In this review, we analyse current data about the possible causes of pili tori and discuss the underlying conditions. Available data on the management of pili torti are presented.

## 2. Inherited Pili Torti

Inherited pili torti may be categorized as: classic early onset (Ronchese type), late onset (Beare type), and pili torti associated with genetic diseases or syndromes [2].

### 2.1. Isolated Pili Torti

#### 2.1.1. Early Onset (Ronchese) Type

The classic (Ronchese) form is an autosomal dominant or recessive condition, beginning in the early childhood [2,13]. The disease onset is between the third month and third year of life. Girls with blond hair are most commonly affected. In early onset pili torti, clusters of twists of hair are usually detected. The condition often improves with age, especially after puberty [2,13].

#### 2.1.2. Late Onset (Beare) Type

Late onset type is an autosomal dominant disorder, typically occurring after puberty. It is more commonly observed in individuals with dark hair. Contrary to the early onset type, the twists of hair are usually single in the late onset type [2,13].

### 2.2. Pili Torti in Genetic Diseases or Syndromes

The list of genetic diseases and syndromes associated with pili torti is presented in Table 1. The most common conditions are described below.

#### 2.2.1. Menkes Disease

Menkes disease, also known as kinky hair disease, is a rare neurodegenerative disease with X-linked recessive inheritance [45]. It is caused by different mutations in the ATPase Copper Transporting Alpha (ATP7A) gene, that result in the failure of copper absorption in the intestines followed by copper deficiency [46].

Pili torti is the most characteristic hair shaft defect in Menkes disease. The twists of hair shaft are probably due to the low activity of copper-dependent enzymes, which are important in creating disulfide bonds in hair keratin. Other hair shaft abnormalities are trichoclasis, trichorrhexis nodosa, and trichoptilosis [5].

In patients with Menkes disease, neurodegenerative signs, including seizures, feeding difficulties, hypotonia, and psychomotor retardation usually occur at 2–3 months of age [5]. Other common features are a jowly face with fair complexion, skin and joint laxity, arterial abnormalities, skeletal changes, and urinary tract infections caused by the diverticula of the bladder [5].

In therapeutic management, copper supplementation is the most important [5].

#### 2.2.2. Björnstad Syndrome

Björnstad syndrome is an autosomal recessive or dominant condition caused by a missense mutation in the ubiquinol-cytochrome c reductase complex chaperone (BCS1L) gene [47]. It is characterized by the presence of pili torti and sensorineural hearing loss [47].

In Björnstad syndrome, pili torti develops during the first two years of life [20,21]. It usually affects exclusively the scalp hair. Eyebrows and eyelashes remain unaffected [20,21]. A positive correlation between severity of hair shaft abnormalities and hearing loss has been suggested [20].

#### 2.2.3. Netherton Syndrome

Netherton syndrome is a rare autosomal recessive disorder characterized by a triad of symptoms: congenital ichthyosiform erythroderma, hair shaft abnormalities, and atopic diathesis [48,49]. It is caused by a mutation in the serine protease inhibitor Kazal type 5 (SPINK5) gene, which encodes lymphoepithelial Kazal-type-related inhibitor (LEKTI) [48,49].

Hair shaft abnormalities in Netherton syndrome are probably caused by disturbances in the keratinization process [50]. Pili torti is frequently observed, however trichorrhexis invaginata (“bamboo hair”) is a pathognomonic feature. Other hair shaft defects, such as trichorrhexis nodosa, trichoschisis, and trichoptilosis may also be detected [35,36]. Hair shaft abnormalities in Netherton syndrome tend to improve with age. Complete resolution may occur [51,52]. The presence of hair shaft abnormalities is one of diagnostic criteria of Netherton syndrome, which emphasizes their importance in the diagnostic process [53].

#### 2.2.4. Bazex-Dupré-Christol Syndrome

Bazex-Dupré-Christol syndrome is an X-linked semidominant disorder characterized by follicular atrophoderma, multiple milia, hypotrichosis, hypohidrosis, and an early development of basal cell carcinomas [54,55]. It is caused by mutations in the actin-related protein T1 (ACTRT1) gene [56].

Hair shaft abnormalities may be an initial presentation of Bazex-Dupré-Christol syndrome, and may precede the development of basal cell carcinomas [57]. They are reported in 85% of cases, and include pili torti and trichorrhexis nodosa [58]. Hypotrichosis is usually diffuse in boys, whereas abnormal hair is admixed with normal hair in girls. Eyebrows may also be affected [57].

### 2.3. Pili Torti in Ectodermal Dysplasias

Ectodermal dysplasias include a heterogenous group of inherited disorders, characterized by congenital defects in one or more ectodermal structures and their appendages (hair, teeth, nails, and sweat glands) [59]. Pili torti has been reported in numerous ectodermal dysplasias (Table 2). The selected conditions are described below.

#### 2.3.1. Rapp-Hodgkin Syndrome

Rapp-Hodgkin syndrome is a form of ectodermal dysplasia inherited as an autosomal dominant trait and characterized by anhidrotic ectodermal dysplasia, cleft lip, and cleft palate [80]. It is caused by the mutations in the Tumor Protein P63 (TP63) gene encoding p63 transcription factor [81].

It was hypothesized that hypoplastic adnexal structures, hypoplastic epidermis, folliculitis, atopy, and immunological disturbances in Rapp-Hodgkin syndrome contribute to scalp dermatitis, alopecia, and hair shaft abnormalities [82]. Hair shaft abnormalities in the disease include pili torti and pili canaliculi [74,75]. Scalp hair, eyebrows, eyelashes, and other body hair are affected. Alopecia usually does not affect the occipital and temporal areas [83,84]. Pili torti is one of the most distinctive clinical features in patients with Rapp-Hodgkin syndrome. Thus, it helps to discern it from other types of ectodermal dysplasia with dental abnormalities [85].

Other features of the Rapp-Hodgkin syndrome are narrow nose, small mouth, oligodontia or anodontia, conical teeth, anonychia, hyponychia, narrow or dystrophic nails, ear and ear canal abnormalities, lacrimal duct abnormalities, and genitourinary abnormalities [86,87].

#### 2.3.2. Ankyloblepharon-Ectodermal Defects-Cleft Lip/Palate Syndrome

Ankyloblepharon-ectodermal defects-cleft lip/palate syndrome is a form of ectodermal dysplasia inherited in an autosomal dominant fashion caused by mutations in TP63 gene [60].

Pili torti is a common finding in the disease, observed in up to 59% of cases [60]. Pili trianguli et canaliculi, and irregular indentation and shallow grooves are also commonly detected [60].

Other clinical features in ankyloblepharon-ectodermal defects-cleft lip/palate syndrome include abnormal fibrous strands of tissue that can partially or completely fuse the upper and lower eyelids (ankyloblepharon), mild to severe skin erosions, and cleft palate and/or cleft lip. Scalp erosions are considered as a main cause of morbidity in infants with ankyloblepharon-ectodermal defects-cleft lip/palate syndrome [88].

## 3. Acquired Pili Torti

Acquired pili torti may be associated with numerous dermatological and systemic conditions (Table 3) or may be drug-induced (Table 4).

### 3.1. Pili Torti Associated with Cicatricial Alopecias

The term cicatricial alopecia corresponds to a heterogeneous group of disorders characterized by irreversible destruction of hair follicles with subsequent scarring [109]. It results in abnormal hair shaft formation with the presence of pili torti [7]. To date, data considering pili torti in cicatricial alopecias are limited. However, it may be suggested that the number of pili torti correlates with the severity and duration of inflammatory/fibrosis process.

The clinical and trichoscopic characteristics of cicatricial alopecias associated with pili torti are presented in Table 5.

#### 3.1.1. Pili Torti Associated with Primary Cicatricial Alopecias

##### Lichen Planopilaris

The classic variant of lichen planopilaris is one of the most common forms of primary cicatricial alopecia [110]. The pathogenesis of the disease has not been fully elucidated [109]. However, a misdirected cellular immune response to an unknown antigen in the basement membrane zone, leading to the destruction of follicular stem cells in the bulge region of the hair follicle has mainly been suggested [109].

Pili torti is identified in 31.82–51.9% of patients with lichen planopilaris [89,111]. It is usually present in long-lasting disease [89,111]. Other characteristic trichoscopic features of lichen planopilaris are perifollicular scaling and perifollicular erythema [112].

##### Frontal Fibrosing Alopecia

Frontal fibrosing alopecia is considered as a variant of lichen planopilaris [113,114]. The etiology and the pathogenesis of the disease are still unknown [114]. However, the role of sexual hormones in the development of frontal fibrosing alopecia has been proposed [114].

Pili torti is observed in 71.4% of patients with frontal fibrosing alopecia [89]. Its presence has been also described in the eyebrow area [115]. Similar to the classic variant of lichen planopilaris, perifollicular erythema and perifollicular scaling are the most characteristic trichoscopic features of frontal fibrosing alopecia [116].

##### Discoid Lupus Erythematosus

Discoid lupus erythematosus is the most common subtype of chronic cutaneous lupus erythematosus [114]. 

Pili torti is present in 7.3–14.3% of cases [89,92]. Other characteristic trichoscopic findings of discoid lupus erythematosus include follicular red dots, large yellow dots, and thick arborizing vessels [2].

##### Pseudopelade of Brocq

Pseudopelade of Brocq is a form of lymphocytic primary scarring alopecia. There is still no clear consensus whether the disease is a distinct entity or represents the end stage of any given cicatricial scalp disorder [109,114]. 

Pili torti is observed in 40% of patients with pseudopelade of Brocq [89]. Other trichoscopic findings of the disease are non-specific [89,117,118,119].

##### Folliculitis Decalvans

Folliculitis decalvans is a form of primary neutrophilic cicatricial alopecia [114]. The frequent association with Staphylococcus aureus infections suggests that the disease is caused by an excessive inflammatory response to staphylococcal antigens [109].

Pili torti is identified in 47.1% of patients with folliculitis decalvans, regardless of the severity and stage of the disease [89,120]. Other characteristic trichoscopic features of the disease include hair tufts consisting of 5–20 hairs surrounded by yellowish tubular scaling [117].

##### Dissecting Cellulitis

Dissecting cellulitis is an infrequent form of primary neutrophilic cicatricial alopecia characterized by the occlusion of follicular openings [121].

Pili torti is present in 16.7% of cases [89]. The most characteristic trichoscopic findings of dissecting cellulitis are 3D (soap bubble) yellow dots [117,122].

##### Central Centrifugal Cicatricial Alopecia

Central centrifugal cicatricial alopecia is a form of lymphocyte-predominant cicatricial alopecia [123]. The pathogenesis of the disease has not been fully described. The role of genetic predisposition, use of chemical straighteners, traction hairstyles, bacterial or fungal infections of the scalp have been suggested [109]. 

Pili torti was described in a few cases of central centrifugal cicatricial alopecia [91]. The most specific trichoscopic feature of the disease is peripilar gray/white halo [124].

#### 3.1.2. Pili Torti Associated with Secondary Cicatricial Alopecias

##### Traction Alopecia

Traction alopecia is a form of acquired hair loss that results from persistent, pulling forces on the hair follicles associated with traction-inducing hairstyles [125]. In its early phase, areas of non-scarring hair loss are present. Subsequently, the disease may progress to scarring alopecia [125].

Pili torti is reported in 56% of patients with traction alopecia, and is present during the scarring stage of the disease [93]. Other trichoscopic findings of traction alopecia are non-specific [93].

##### Linear Scleroderma en Coup de Sabre

Linear scleroderma “en coup de sabre”, a form of linear morphea, is one of the cause of secondary cicatricial alopecia.

In linear scleroderma “en coup de sabre”, diffuse distribution of pili torti is observed [95]. Moreover, trichoscopy reveals scattered black dots, broken hairs, and short thick linear and branching tortuous vessels on the periphery of the lesion [95].

### 3.2. Pili Torti in Non-Cicatricial Alopecias

#### Pili Torti in Alopecia Areata

Alopecia areata is an autoimmune form of non-scarring hair loss that may affect any hair-bearing area. It is caused by the infiltration of T helper cells, cytolytic T cells, natural killer cells, and plasmacytoid dendritic cells around the lower part of the hair bulb during the anagen phase, which induces the collapse of the hair follicle immune privilege and hair loss [126].

The pathogenesis of pili torti in alopecia areata has not been described [90]. It may be hypothesized that it results from perifollicular infiltrates that induce pressure on the epithelium and disrupt hair shaft formation. Pili torti in alopecia areata was reported in one study conducted by Park et al. [90] with the frequency 6% and 2% of patients with localized and diffuse hair loss, respectively. The most characteristic trichoscopic features of alopecia areata are exclamation mark hairs, while yellow dots and vellus hairs [2].

### 3.3. Pili Torti in Malignancies

Pili torti was described as the most common trichoscopic feature of the erythrodermic variants of cutaneous T-cell lymphoma present in 81% of cases [98]. It is characterized by high sensitivity and specificity of 81% and 93%, respectively [98]. It was hypothesized that in cutaneous lymphomas, pili torti results from folliculotropic inflammation without or with the mucinous degeneration of the hair follicle, which induces pressure on the epithelium, and, thus, affects hair shaft formation [98].

Moreover, one case report described a patient with scalp metastases of breast cancer, with the presence of pili torti on trichoscopic examination [97].

### 3.4. Drug-Induced Pili Torti

Associations between pili torti and numerous drugs were described in the literature [104,105,106,107,108].

Pili torti is most commonly described after the use of epidermal growth factor receptor inhibitors. In an animal model, it was reported that epidermal growth factor receptor inhibitors impair DNA integrity and lead to apoptosis of both keratinocytes (interfollicular epidermis, outer root sheath, and matrix) and non-proliferative, differentiated cells of the hair shaft [127]. Inhibition of epidermal growth factor receptor pathways also interferes with transcription factor expression. Thus, it impairs proper differentiation cues for hair shaft cells, resulting in loss of the medulla layer. Moreover, in mice harboring a disruption of the epidermal growth factor receptor-allele it was showed that hair follicles fail to enter catagen, and remain in an aberrant anagen state [104]. Subsequently, the thinning or loss of the outer and inner root sheaths, together with perifollicular inflammation and fibrosis are detected. In a case series of patients treated with erlotinib, histological examination of the scalp showed irregular thinning of the outer root sheath and disintegration of the inner root sheath [104].

Other drugs which may induce pili torti are oral retinoids, sodium valproate, and carbamide perhydrate.

Retinoic acid plays an important role in hair follicle formation and patterning through the regulation of the homeobox genes [128]. It was suggested that retinoids influence the keratinization of the inner root sheaths of the hair follicle in the anagen phase, which leads to pili torti formation [129].

The mechanism of pili torti formation after valproate use is unclear. It may be associated with valproate chelating properties (copper, zinc, and magnesium) as well as the inhibition of metalloproteins [130].

Carbamide peroxide is a source of hydrogen peroxide, which affects the hair shaft by oxidation and decomposition of cysteine (which accounts for 20% of the amino acids of the hair keratin) as well as the formation of cysteic acid. Moreover, carbamide peroxide destroys the majority of the disulfide bridges of hair keratin [108].

### 3.5. Other Secondary Causes of Pili Torti

Pili torti was also described in other systemic conditions, such as anorexia nervosa [100,101], malnutrition [103], cataracts [99], and chronic graft-vs.-host disease [102].

## 4. Diagnosis

The diagnosis of pili torti is based on trichoscopic and microscopic examination.

**Figure 2:**
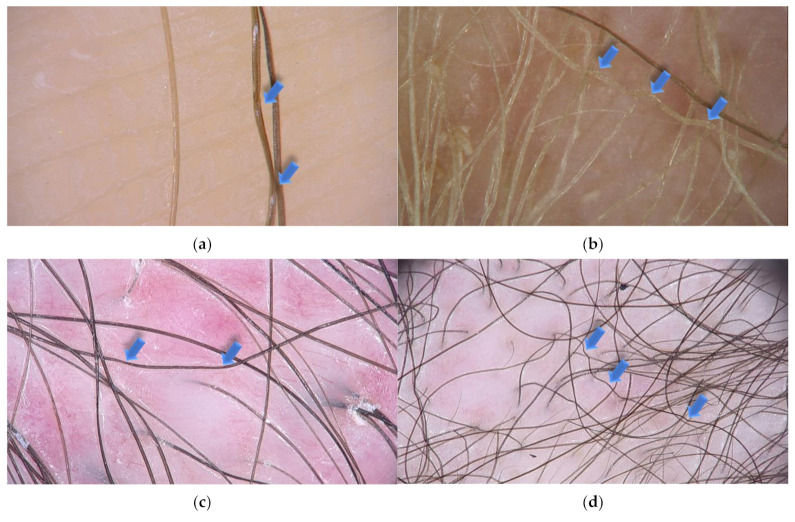
Trichoscopy shows pili torti (blue arrows) in various local and systemic conditions. (**a**) Pili torti in patient with late onset (Beare) type with single twists of hair (×70); (**b**) pili torti in patient with ectodermal dysplasia with the presence of multiple twist of hair (×50); (**c**) pili torti in patient with lichen planopilaris. Perifollicular scaling and milky-red areas are also presented (×20); (**d**) numerous pili tori in patient with mycosis fungoides (×20).

Trichoscopy, hair and scalp dermoscopy, is a rapid technique that is useful in the diagnosis of scalp and hair diseases as well as genetic disorders, including ectodermal dysplasias [10,131]. It can be performed with a manual dermoscope (10 magnification) or a videodermoscope (20–1000 magnification) [132]. This noninvasive method replaced light microscopy, which required pulling of multiple hairs for investigation. This is particularly burdensome in cases, where only few hairs might be affected [9]. In pili torti, low magnification trichoscopy reveals the hair shafts bent at different angles and at irregular intervals. Regular twists of the hair shaft along the long axis are observed at high magnification (Figure 2).

Microscopic examination shows groups of three or four regularly spaced twists at irregular intervals along the shaft [10].

Genetic diseases and syndromes should be excluded in every patient with pili torti. The search of other signs of underlying conditions should be performed in the case of acquired pili torti.

## 5. Treatment

There is no specific treatment of pili torti. The avoidance of trauma to the hair is recommended. Other forms of management include sleeping on a satin pillowcase, avoiding excessive grooming, braiding, heat treatments, and dying. Gentle shampoos may be beneficial [104,133].

Congenital pili torti may improve spontaneously after puberty. Drug-induced cases tend to resolve after the discontinuation of the offending agent [105,106]. In regard to acquired pili torti, the treatment of the underlying condition is most important.

Efficacy of pharmacological treatment in pili torti is limited [26]. Topical minoxidil has been suggested as a beneficial therapeutic option for patients with hair shaft abnormalities with increased fragility. However, it only has an impact on hair density and does not induce a causal treatment.

## 6. Conclusions

Pili torti is a rare condition, which may be associated with numerous congenital or acquired conditions. In every case of pili torti, the identification of the underlying disorder determines the therapeutic approach and prognosis.

## Figures and Tables

**Table 1:** Pili torti in genetic diseases and syndromes.

Disease/Syndrome	Genetic Defect, Inheritance	Other Clinical Findings	Other Hair Shaft Abnormalities
Abnormal hair, joint laxity, and developmental delay [14,15]	HEPHL1 gene (AR)	growth and developmental delay, joint laxity, neurologic abnormalities	trichorrhexis nodosa
Acrofacial dysostosis, Palagonia type [16]	NK (AD)	short stature, vertebral anomalies, syndactyly, oligodontia, cleft lip	-
Arginosuccinic aciduria [17]	ASL gene (AR)	lethargy, vomiting, seizures, cerebral edema, hepatomegaly	trichorrhexis nodosa
Autosomal recessive ichthyosis with hypotrichosis [18]	ST14 gene (AR)	lamellar ichthyosis, follicular atrophoderma, hypohidrosis	dysplastic hair, pili bifurcati
Bazex-Dupre-Christol syndrome [19]	ACTRT1 gene (XD)	follicular atrophoderma, multiple basal cell carcinomas, milia, hypohidrosis	trichorrhexis nodosa
Björnstad syndrome [20,21]	BCS1L gene (AR, AD)	sensorineural hearing loss	-
Citrullinemia [22]	ASS1 gene (AR)	hyperammonemia, lethargy, poor feeding, vomiting, high intracranial pressure, scaly skin eruption	trichorrhexis nodosa
Congenital disorder of glycosylation, type Ia [23]	PMM2 gene (AR)	hypotonia, strabismus, cerebellar hypoplasia, seizures, mental and physical retardation, hepatomegaly, liver fibrosis, fat pads, ‘orange peel’ skin	trichorrhexis nodosa
Congenital erythropoietic porphyria [24]	UROS gene (AR)	severe skin photosensitivity (scarring, blistering), erythrodontia, reddish-colored urine, anemia	-
Congenital hypotrichosis with juvenile macular dystrophy [25]	CDH3 gene (AR)	retinal degeneration	-
Conradi-Hünermann syndrome [26]	EBP gene (XD)	short stature, asymmetric short limbs, vertebral malformations, hip dysplasia, chondrodysplasia punctata, follicular atrophoderma, abnormal nails, craniofacial anomalies, cataracts	-
Crandall syndrome [27]	NK (AR)	neurosensory deafness, hypogonadism with decreased levels of luteinizing hormone and growth hormone	-
Giant axonal neuropathy [28]	GAN gene (AR)	progressive sensorimotor peripheral neuropathy, axonal loss, optic atrophy, ophthalmoplegia, skeletal deformations	trichorrhexis nodosa
Hypotrichosis 6 [29,30]	DSG4 gene (AR)	hyperkeratotic follicular papules, erythema, scaling, dry skin	monilethrix-like hair, trichoschisis, trichorrhexis nodosa-like defects, tapered hair
Laron syndrome [31]	GHR gene (AR)	short stature, obesity, facial dysmorphism, hypogenitalism, elevated serum growth hormone, undetectable or low serum insulin-like growth factor 1	-
Marie Unna hypotrichosis [32]	U2HR gene (AD)	-	-
McCune-Albright syndrome [33]	GNAS1 gene (not inherited)	polyostotic fibrous dysplasia, cafe-au-lait skin pigmentation, multiple endocrine dysfunction	-
Menkes disease [5]	ATP7A gene (XR)	growth retardation, vascular, neurological, and skeletal abnormalities, pale skin	trichoclasis, trichorrhexis nodosa, trichoptilosis
Mitochondrial diseases [34]	all modes of inheritance can be expected	mental retardation, failure to thrive, hypotonia, hypoparathyroidism	longitudinal grooving with cuticle loss
Netherton syndrome [35,36]	SPINK5 gene (AR)	congenital erythroderma, atopic manifestations, increased IgE level	trichorrhexis I vaginata, trichorrhexis nodosa, trichoschisis, trichoptilosis
Occipital horn syndrome [37]	ATP7A gene (XR)	tallness, pectus excavatum, dorsal kyphosis, occipital horn exostoses, joint laxity, loose skin, decreased serum copper and ceruloplasmin	-
Olmsted syndrome [38]	TRPV3 gene (AD)	constriction of digits (‘pseudoainhum’), mutilating palmoplantar keratoderma, onychodystrophy, periorificial keratotic plaques	trichorrhexis nodosa
Peeling skin syndrome [39]	CDSN gene (AR)	superficial patchy peeling of the entire skin, erythroderma, atopy, nail anomalies	trichorrhexis invaginata-like changes, monili-form hair shaft diameter reductions, irregular hair shaft torsions
Salti-Salem syndrome [40]	NK (AD)	hypogonadotropic hypogonadism	-
Steatocystoma multiplex [41]	KRT17 gene (AD)	subcutaneous cysts	pili canaliculi
Tricho-hepato-enteric syndrome [42]	TTC37, SKIV2L genes (AR)	low birth weight, failure to thrive, facial dysmorphism, diarrhoea, liver disease	trichorrhexis nodosa, aniso- and poilkilotrichosis
Trichothiodystrophy, photosensitive [43,44]	ERCC2, XPD genes (AR)	mental and physical retardation, short stature, facial dysmorphism, ichthyosis, photosensitivity, ocular abnormalities	Pili annulati (‘tiger-tail’ hair), trichoschisis, trichorrhexis nodosa

AD, autosomal dominant; AR, autosomal recessive; NK, not known; XD, X-linked dominant; XR, X-linked recessive.

**Table 2:** Pili torti in ectodermal dysplasias.

Disease/Syndrome	Genetic Defect, Inheritance	Other Clinical Findings	Other Hair Shaft Abnormalities
Ankyloblepharon-ectodermal defects-cleft lip and palate syndrome [60]	TP63 gene (AD)	hypoplastic maxilla, palmoplantar hyperkeratosis, dystrophic nails, cleft lip/palate, dental anomalies, ankyloblepharon, lacrimal duct atresia, auricular abnormalities	pili canaliculi, trichoclasis, trichorrhexis nodosa, pili annulati, pili triangulati
Basan syndrome [61]	SMARCAD1 gene (AD)	neonatal blisters and milia, adermatoglyphia, traumatic blistering and fissuring, hypohidrosis	-
Cleft lip/palate-ectodermal dysplasia syndrome [25]	PVRL1 gene (AR)	mental retardation, facial dysmorphism (protruding and malformed ears, micrognathia, bilateral cleft lip/palate), syndactyly, palmoplantar keratoderma, hypohidrosis, teeth, and nail anomalies	-
Ectodermal dysplasia 4, hair/nail type [62]	KRT85, KRT74, HOXC13 genes (AR)	congenital nail dystrophy	-
Ectodermal dysplasia with corkscrew hairs [26,63]	NK (AR)	facial dysmorphism, cleft lip/palate, scalp keloids, follicular plugging, keratosis pilaris, xerosis, eczema, palmoplantar keratodermia, cutaneous syndactyly, onychodysplasia, teeth abnormalities	-
Ectodermal dysplasia with syndactyly [25]	PVRL-4 gene (AR)	highly arched palate, teeth abnormalities, syndactyly, hypoplastic nails, dry skin with hyperkeratosis	-
Ectrodactyly, ectodermal dysplasia, and cleft lip/palate syndrome 3 [64]	EEC3 gene (AD)	hearing loss, cleft lip/palate, dysplastic teeth, ectrodactyly, syndactyly, nail dystrophy, hypopigmentated skin, hyperkeratosis, skin atrophy, genitourinary anomalies	pseudomoniletrix, pili canaliculi, longitudinal grooving, trichothiodystrophy
Goltz syndrome [65]	PORCN gene (XD)	cleft palate, syndactyly, polydactyly, skin atrophy, telangiectasia, herniation of fat, papillomas, nail and teeth anomalies, ocular anomalies (coloboma of iris and choroid, strabismus, microphthalmia)	atrophic hair with reduced diameters, flattened hair shafts, trichorrhexis nodosa, pili trianguli et canaliculi
Hidrotic ectodermal dysplasia [66,67,68]	GJB6 gene (AD)	short stature, clubbed digits, palmoplantar hyperkeratosis, hyperpigmentation, nail dystrophy, cataract, photophobia, strabismus	trichorrhexis nodosa, trichoptilosis, pili bifurcati, variable diameter, damaged cuticles, irregular helical twists, pili canaliculi
Hypohidrotic Ectodermal Dysplasia [12]	EDA1/EDAR, EDARADD, WNT10A genes (XR, AR, AD)	facial dysmorphism (prominent forehead, thick lips, flattened nasal bridge), teeth abnormalities, hypohidrosis	trichorrhexis nodosa, pili bifurcati, variable shaft thickness
Hypotrichosis-osteolysis-periodontitis-palmoplantar keratoderma syndrome [25]	NK gene (AD)	onychogryphosis, acroosteolysis, linear or reticular palmoplantar keratoderma and erythematous, psoriasis-like skin lesions, periodontitis, premature teeth loss, lingua plicata, ventricular tachycardia	pili annulati
Oculo-dento-digital syndrome [69,70]	GJA1 gene (AD, AR)	facial dysmorphism (narrow, pinched nose, hypoplastic alae nasi, prominent columella, narrow nasal bridge), microphthalmia, microdontia, syndactyly, camptodactyly, clinodactyly, brittle nails	“tiger tail” aspect, monilethrix, pili annulati
Pachyonychia congenita-2 [71,72,73]	KRT17 gene (AD)	palmoplantar hyperkeratosis, nail dystrophy, hyperhidrosis, cystic lesions (steatocystoma multiplex, pilosebaceous cysts), folliculitis, natal teeth	-
Rapp-Hodgkin syndrome [74,75]	TP63 gene (AD)	short stature, hypohidrosis, facial dysmorphism (narrow nose, small mouth, cleft lip, hypoplastic maxilla, prominent, malformed auricles), dysplastic nails, teeth abnormalities, chronic epiphora	pili canaliculi
Reeds syndrome [76]	NK (AD)	lobster claw deformity, nasolacrimal obstruction, cleft lip/palate, teeth abnormalities	-
Salamon syndrome [77]	NK (AR)	everted lower lip, teeth abnormalities, protruding ears	-
Schöpf-Schulz-Passarge syndrome [78]	WNT10A gene (AR)	Palmoplantar keratoderma, nail dystrophy, hypodontia, eyelid cysts	-
Trichodysplasia-xeroderma [79]	NK (AD)	dry skin	trichorrhexis nodosa

AD, autosomal dominant; AR, autosomal recessive; NK, not known; XD, X-linked dominant; XR, X-linked recessive.

**Table 3:** Conditions associated with acquired pili torti.

Conditions Associated with Acquired Pili Torti
lichen planopilaris [89];
frontal fibrosing alopecia [89];
alopecia areata [90];
central centrifugal cicatricial alopecia [91];
discoid lupus erythematosus [89,92];
dissecting cellulitis [89];
folliculitis decalvans [89];
pseudopelade of Brocq [89];
traction alopecia [93];
linear scleroderma en coup de sabre [94,95,96];
repetitive trauma [9];
scalp metastasis of breast cancer [97];
cutaneous T-cell lymphoma [98];
acne conglobate [99];
anorexia nervosa [100,101];
graft-vs.-host disease [102];
hair transplantation [9];
malnutrition [103];
systemic sclerosis [24];
cataracts [99].

**Table 4:** Drugs related to pili torti formation.

Drugs Associated with Pili Torti
epidermal growth factor receptor inhibitors [104,105];
oral retinoids [106];
sodium valproate [107];
carbamide perhydrate [108].

**Table 5:** The clinical and trichoscopic characteristic of cicatricial alopecias associated with pili torti.

Disease	Epidemiology	Clinical Features	Trichoscopy
Lichen Planopilaris	women 40–60 years of age	multifocal, confluent areas of hair loss with perifollicular hyperkeratosis and erythema at the periphery; the vertex and the parietal area are most commonly affected	perifollicular scaling, hair casts, perifollicular erythema, white dots, white and milky red areas, loss of follicular openings
Frontal Fibrosing Alopecia	post-menopausal women	recession of the frontotemporal hairline, eyebrow loss	perifollicular erythema, perifollicular scaling, hair casts, white areas, loss of follicular openings
Discoid Lupus Erythematosus	women 20–40 years of age	well-demarcated annular or oval plaques with follicular plugging, erythema, telangiectasia, scaling, dyspigmentation	follicular red dots, large yellow or yellow-brown dots, “red spiders on yellow dots”, scattered brown discoloration, white and milky red areas, loss of follicular openings
Pseudopelade of Brocq	middle-aged white women	asymptomatic, asymmetrical, white, or porcelain-white patches involving the vertex or parietal area	white dots, white and milky-red areas, loss of follicular openings, variations in hair diameter
Folliculitis Decalvans	young to middle-aged adult men of African descent	tender, recurrent papulo-pustular lesions on the vertex and occipital area	hair tufts consisting of 5–20 hair surrounded by yellowish tubular scaling, starburst sign, coiled capillary loops, white and milky-red areas, loss of follicular openings
Dissecting Cellulitis	young men of African descent	perifollicular pustules, painful nodules, abscesses with sinus tracts involving the vertex and occipital area	3D yellow dots, yellow structureless areas, black dots, pinpoint-like vessels with whitish halo, white areas, loss of follicular openings
Central Centrifugal Cicatricial Alopecia	middle-aged women of African descent	scarring hair loss initially involving the vertex or crown of the scalp and slowly progressing peripherally	peripilar gray/white halo, perifollicular scaling, loss of follicular openings
Traction Alopecia	women and children of African descent	hair loss and thinning, pustules, inflammatory papules; may progress to scarring alopecia	perifollicular erythema, hair thinning, focal decrease in hair density, honeycomb pattern, pinpoint white dots, irregular white patches
Linear Scleroderma en Coup de Sabre	Children and women within the first two decades of life	single erythematous or violaceous linear indurated plaque, progressing to hyperpigmented or hypopigmented streak on the forehead	scattered black dots, broken hairs, short thick linear and branching tortuous vessels on the periphery of the lesion, white areas, loss of follicular openings

## Data Availability

Not applicable.
